# *In Silico* Syndrome Prediction for Coronary Artery Disease in Traditional Chinese Medicine

**DOI:** 10.1155/2012/142584

**Published:** 2012-04-10

**Authors:** Peng Lu, Jianxin Chen, Huihui Zhao, Yibo Gao, Liangtao Luo, Xiaohan Zuo, Qi Shi, Yiping Yang, Jianqiang Yi, Wei Wang

**Affiliations:** ^1^Institute of Automation, Chinese Academy of Sciences, Beijing 100190, China; ^2^Beijing University of Chinese Medicine, 11 Bei San Huan Dong Lu, ChaoYang District, Beijing 100029, China

## Abstract

Coronary artery disease (CAD) is the leading causes of deaths in the world. The differentiation of syndrome (ZHENG) is the criterion of diagnosis and therapeutic in TCM. Therefore, syndrome prediction *in silico* can be improving the performance of treatment. In this paper, we present a Bayesian network framework to construct a high-confidence syndrome predictor based on the optimum subset, that is, collected by Support Vector Machine (SVM) feature selection. Syndrome of CAD can be divided into asthenia and sthenia syndromes. According to the hierarchical characteristics of syndrome, we firstly label every case three types of syndrome (asthenia, sthenia, or both) to solve several syndromes with some patients. On basis of the three syndromes' classes, we design SVM feature selection to achieve the optimum symptom subset and compare this subset with Markov blanket feature select using ROC. Using this subset, the six predictors of CAD's syndrome are constructed by the Bayesian network technique. We also design Naïve Bayes, C4.5 Logistic, Radial basis function (RBF) network compared with Bayesian network. In a conclusion, the Bayesian network method based on the optimum symptoms shows a practical method to predict six syndromes of CAD in TCM.

## 1. Introduction

 Coronary artery disease (CAD), which is a narrowing of the small blood vessels that supply the heart with blood, oxygen, and nutrients, is the most common cardiovascular disease (CVD). It is the leading cause of death in the world. According to the newest World Health Organization reports, an estimated 17.3 million people died from CVD in 2008, representing 30% of all global deaths [1]. CAD is responsible for a large proportion of CVD, accounting for an estimated 7.3 million (12.57%) [2].

CAD is caused by many factors such as genetics, the environment, harmful use of alcohol, unhealthy diet, tobacco, and others. In western medicine, CAD is treated by surgical operation, pharmaceutical drugs, physical activity, and other interventional therapies. These achievements typically lead to good outcomes by decreasing rates of death related to CAD. However, these methods generally focus on the structure and function of the heart, but ignore differences in systematic functions, curative reaction, and the individual. Since western medicine faces problems such as high cost and significant side effects, Traditional Chinese Medicine (TCM) can be a complementary alternative to overcome these defects. In TCM, CAD belongs to the scope of chest heartache and cardiodynia [3]. TCM, which has a history of thousands of years, makes significant contributions to people worldwide, especially in Asia. The TCM approach is fundamentally different from that of western medicine [4]. In TCM, the human body is based on the holistic understanding of the universe and is recognized by system discrimination in a cybernetic way [5]. Zheng (syndrome) is the key pathological principle of TCM. All diagnostic and therapeutic methods in TCM are based on the differentiations of syndrome (ZHENG), and this concept has been used for thousands of years in China [6, 7]. A syndrome is constituted by a set of symptoms, including subjective feeling and objective signs. It is the integrative response of the body state in the body's internal and external environment. In the process of disease development, syndromes changes dynamically with rise or fall of corresponding occurrence of evidence. A disease is the nature of a comprehensive reflection of one or more syndromes in the different stages of pathology. In the process of development of CAD, syndromeprediction *in silico *is a potentially logical choice for prevention and treatment.

In order to achieve an effective and objective standard of syndrome prediction, many researchers have used a data mining approach to construct the classifier for the TCM dataset [8, 9]. Syndrome prediction is regarded as supervised classification analysis: the class label is the diagnosis, and features are the symptoms of the patient. Because clinical diagnosis datacontain irrelevant features and noise, the identification of the related symptoms is an important problem in syndrome prediction aside from classifying the syndrome.

In recent years, there has been remarkable progress in thesyndrome predictionof TCM. Data have focused on two aspects: feature selection (symptom selection) and syndrome prediction (syndrome classification). Jie et al. investigated syndrome factors of CAD by using the support vector machine (SVM) method on the basis of 15 typical medical records from prominent TCM doctors. Eight syndromes were drawn, including blood stasis, turbid phlegm, Qi deficiency, Yang insufficiency, Yin deficiency, inner heat, blood deficiency, and Qi stagnation [10]. Li et al. compared the cold and hot syndrome networks through literature searches and found that hormones are predominant in the Cold ZHENG network, immune factors are predominant in the Hot ZHENG network, and these two networks are connected by neurotransmitters [6]. Zhou et al. developed a clinical data warehouse system including medical knowledge discovery and TCM clinical decision support to use variousclassification methods, namely, machine SVM decision tree and Bayesian network, to look at syndrome differentiation [11]. Chen et al. proposed a novel pattern discovery algorithm based on revised mutual information to discover syndromesfor chronic renal failure [12]. In regards to CAD, Liu et al. designed standardization scale on inquiry diagnosis and constructed this diagnostic model by using the method of multilabel learning [3]. In addition, many techniques of data mining are applied to syndromes in TCM [9, 13–26]. 

Though many achievements have been made in syndrome prediction, there are still some problems left, which deserve discussion [8]. Our research is focused on discovering symptoms of TCM, and lab-measured indexes are rarely included. The characteristics of CAD syndrome are usually not considered when the classifier is built. First, we used symptoms including TCM and western symptoms for identifying syndromes of CAD. Second, we constructed six predictors to classify six syndromes of CAD. Third, the related symptoms were selected based on characteristics of syndromes of CAD and were placed into three classes: sthenia, asthenia, or both.

In this paper, 987 CAD cases were used for selecting related symptoms and building the predicting model of CAD syndrome. Based on symptoms, we propose a syndrome prediction method which integrates SVM feature selection and Bayesian network classifier to improve the predictive performance of the classifier.

The rest of this paper is organized as follows.  Section 2 describes materials and methods including data description, preprocessing and symptom selection method, syndrome prediction method. Experimental results and discussions are shown in Section 3.  Section 4 draws conclusions from this paper.

## 2. Material and Methods

### 2.1. Material

In this paper, the cases were collected from two provinces including 5 clinical centers from June 2005 to October 2008, where patients who suffered from CAD were surveyed. Each patient was diagnosed by western doctors by means of coronary artery angiography.

Inclusion criteria are as follows [24].

1. Each case must have been diagnosed with CAD defined by the American College of Cardiology (ACC) together with American Heart Association (AHA) in 2002.
2. Each case was verified by coronary artery angiography as having at least one branch of the coronary artery main branch with stenosis larger than 70% or coronary artery left diameter stenosis greater than 50%.
3. Each case must have included an attached informed consent signed by each patient.
4. Each patient was greater than 35 years of age.

In western medicine, the diagnosis of patients was in accordance with the “Guidelines for the diagnosis and management of chronic angina pectoris, unstable angina pectoris, and non-ST elevation myocardialinfarction” released by the ACC/AHA, and “Recommendation about Diagnosis of Diagnosing Unstable Angina Pectoris” released by Chinese Society of Cardiology in 2000. In TCM, syndrome diagnosis was in accordance with the foundation theory of TCM. For example, the diagnosis of blood stasis was judged by “Standard of Blood Stasis Diagnosis” (1986.11, Guangzhou); the diagnosis of deficiency was treated by “Standard of TCM Syndrome Differentiation of Deficiency” (1986.5); the diagnosis of turbid phlegmwas decided by “Classification Code of TCM Diseases”; the others depended on the teaching materials (“*Diagnosis of TCM*”).

There were two exclusion criteria [24]:

1. any patient with acute ST-segment elevation myocardial infarction, and
2. any patient who also suffers from concomitant serious diseases such as liver orkidney disease.

Each symptom has four levels: none, light, middle, and severe. Each case was diagnosed as a syndrome by experienced TCM experts. Each symptom was considered a feature; the diagnosed syndrome was taken as a response.

In total, we evaluated 1,008 cases of patients, including the diagnosis results of western medicine and TCM, and over 100 symptoms of both western medicine and TCM. Data were compiled according to the characteristics of syndromes of CAD, sthenia and asthenia syndromes follow CAD. In regards to the foundation and practice of TCM, sthenia syndromes include Qi stagnation, blood stasis, cold coagulation, phlegm turbidity, heat accumulation, water retention, and dampness pathogen; asthenia syndromes include Qi deficiency, blood deficiency, Yin deficiency, Yang deficiency, heart deficiency, liver deficiency, spleen deficiency, kidney deficiency, and lung deficiency.

### 2.2. Data Preprocessing

**Figure 1:**
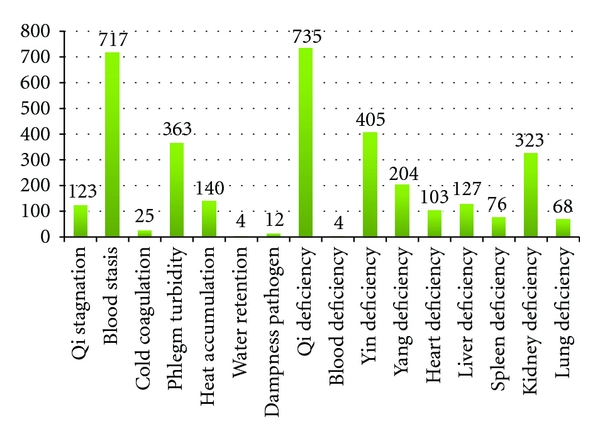
Histogram of syndromes of TCM.

In every case, there were over 70 diagnostic symptoms in TCM and above 30 lab-measured symptoms in the western medicine information. For TCM diagnosis, there was Qi stagnation, blood stasis, cold coagulation, phlegm turbidity, heat accumulation, Qi deficiency, Yin deficiency, and so on. A histogram of syndromes of TCM diagnosis results is shown in Figure 1.

In the process of medical surveys, there inevitably exists missing data. Cases were discarded if the missing data frequency rate of it symptom was more than 70%. Some symptoms which were not treated by data mining technique were removed. If its syndrome was not in the top six syndromes, the case was discarded. Overall, there were 113 features including 78 TCM symptoms and 35 lab-measured indexes. Details of the symptoms are shown in Table 1.

### 2.3. Method of Syndrome Prediction of CAD

In general, syndrome prediction of CAD included the symptom selection phase and syndrome prediction phase. Symptom selection was regarded as the problem of feature selection, and syndrome prediction was regarded as supervised pattern classification in data mining fields. In the feature selection phase, mingling symptoms including TCM symptoms and western symptoms were selected to be used as feature of the syndrome prediction model. In the syndrome prediction phase, every case was classified as blood stasis, phlegm turbidity, Qi deficiency, Yin deficiency, Yang deficiency, and kidney deficiency based on the syndrome prediction model.

#### 2.3.1. Symptom Selection

Symptoms are essential to diagnose CAD for everyone from TCM doctors to western medicine doctors. Therefore, a strong predicting model of syndrome is based on key symptoms. In this phase, we investigated which symptoms influence the predicted syndromes most. We propose two feature selection methods to discover critical symptoms. In this paper, we design SVM and Markov blanket feature selection methods to identify the optimal symptom subset.

SVMs have been an acknowledged tool with high accuracy and efficiency for data classification. The basic idea is to map data into a high dimensional space and find a separating hyperplane with the maximal margin [27]. Given the training vectors *x*_*k*_ ∈ *R* ^*n*^, *k* = 1,2,…, *m* in two classes, and a vector of labels *y* ∈ *R* ^*m*^ such that  *y*_*k*_ ∈ {−1, 1}, SVM solves a quadratic optimization problem [28, 29]:

(1) $$\begin{array}{cc} \underset{\omega ,b,\xi }{\min} & \;\frac{1}{2}\omega^{T}\omega +C\overset{m}{\underset{k=1}{\sum }}\xi_{k}\\ \text{subject}\;\text{to} & \;y_{k}\left(\omega^{T}\phi \left(x_{k}\right)+b\right)\geq 1-\xi_{k}\\ & \;\xi_{k}\geq 0,\;k=1,\ldots ,m, \end{array}$$

where training data are mapped to a higher dimensional space by the function *ϕ*, and *C* is a penalty parameter on the training error. For any training instance *x*, the decision function (predictor) is

(2) $$f\left(x\right)=\mathrm{sgn}\left(\omega^{T}\phi \left(x\right)+b\right).$$

Generally, the nonlinear mapping function *ϕ*(·) is represented by a kernel function  *k*(*x*, *x*′) = *ϕ*(*x*)^*T*^ *ϕ*(*x*′). Several kernels are commonly used such as Gaussian kernel, polynomial kernel, spline kernel, and RBF kernel.

Together with penalty function or optimization objective, SVM can be exploited to select appropriate features or optimal feature groups. As for the feature selection problem, there are two alternative situations [30]: (1) given a fixed *p* ≪ *n* (number of features much less than dimension of feature space), find the *p* features that gives the smallest expected generalization error, or (2) given a maximum allowable generalization error, find the smallest *p*. The former situation will be discussed below, while the latter one can always be formulated as the dual of the former.

One may distinguish between the two types of methods to solve the problem of filter and wrapper methods [31]. The filter method actually performs a procedure of subtractive iterations which removes the least relevant feature iteratively [32]. The wrapper method, on the other hand, is a searching process which starts from a null feature set and chooses the best feature into the feature set in each iteration [33].

Several existing strategies have been combined with SVM for feature selection. Given training vectors *x*_*k*_, *k* = 1,2,…, *m*, if the positive and negative instances are *n*_+_ and *n*_−_, respectively, then the *F*-score of the *i*th feature is defined as

(3) $$\begin{array}{cc} F\left(i\right)= & \frac{{\left({\overset{̅}{x}}_{i}^{\left(+\right)}-{\overset{̅}{x}}_{i}\right)}^{2}+{\left({\overset{̅}{x}}_{i}^{\left(-\right)}-{\overset{̅}{x}}_{i}\right)}^{2}}{\left(1/\left(n_{+}-1\right)\right)\sum_{k=1}^{n_{+}}{\left(x_{k,i}^{\left(+\right)}-{\overset{̅}{x}}_{i}^{\left(+\right)}\right)}^{2}+\left(1/\left(n_{-}-1\right)\right)\sum_{k=1}^{n_{-}}{\left(x_{k,j}^{\left(-\right)}-{\overset{̅}{x}}_{i}^{\left(-\right)}\right)}^{2}}, \end{array}$$

where ${\overset{̅}{x}}_{i}$, ${\overset{̅}{x}}_{i}^{\left(+\right)}$, ${\overset{̅}{x}}_{i}^{\left(-\right)}$ are the average of the *i*th feature of the whole, positive, and negative data sets, respectively; *x*_*k*,*i*_^(+)^ is the *i*th feature of the *k*th positive instance, and *x*_*k*,*i*_^(−)^ is the *i*th feature of the *k*th negative instance.

We selected features with high *F*-scores and then applied SVM for training/prediction. The procedure was as follows [34].

1. Calculate *F*-score of every feature.
2. Pick possible thresholds as cutoffs for *F*-scores.
3. For each threshold, complete the following:
  - drop features with *F*-scores below this threshold,
  - randomly split the training data into *X*_train_ and *X*_valid_,
  - let *X*_train_ be the new training data. Use the SVM procedure to obtain a predictor; use the predictor to predict *X*_valid_,
  - repeat the steps above five times and then calculate the average validation error.
4. Choose the threshold with the lowest average validation error.
5. Drop features with *F*-scores below the selected threshold. Then apply the SVM procedure.

Finally, the features with efficient prediction power were selected.

Compared with SVM feature selection, we also designed Markov blanket feature selection which was firstly proposed by Koller and Sahami in 1996 [35]. A Markov blanket of a target attribute T renders it statistically independent from all the remaining attributes. That is, given the values of the attributes in the Markov blanket, the probability distribution of T is completely determined, and knowledge of any other variable(s) becomes superfluous [36]. Based on their work, several algorithms were proposed to find the optimal feature subset. Cui et al. [37] proposed an approximate feature selection algorithm based on the Markov blanket. They used Chi-Square tests and *P* values to scale the independence between features. For computational simplicity, they constrained the size of the Markov blanket to 1. Fi was declared a Markov blanket of fj when fi had a high correlation with class C and fj was more independent with class C given fi. Zhu et al. [38] proposedan information gain based on the Markov blanket feature selection algorithm: MBEGA. They defined fi to be a Markov blanket of fj on the condition that fi gives more information to class than fj, and fj gives more information to fi than to class C. Compared with MBEGA, MBFS is more in line with the idea of Markov blanket and has a more comprehensive and profound base of information theory.

#### 2.3.2. Syndrome Prediction

Syndrome prediction is important for doctors. In this study we presented a Bayesian network framework to construct a high-confidence syndrome predictor by integrating a comprehensive list of mingling symptoms. In fact, it is a classification that is a basic task in data analysis and pattern recognition that requires construction of a classifier, that is, a function that assigns a class label to instances described by a set of features [39].

Bayesian network, which is one of the most effective classification method for graphically representing and processing feature interdependencies, represents a joint probability distribution over a dataset [39, 40]. Bayesian network is directed acyclic graphs (DAG) that allow for efficient and effective representation of joint probability distributions. In this paper, we constructed a Bayesian network structure to simulate the data modelbased on 897 cases. The nodes in the network were predetermined, one for each symptom or syndrome. The network structures are learned by searching through the space of possible sets of edges, estimating the conditional probability stables for each set, and computing the log-likelihood of the resulting network based on the data as a measure of the network's quality [41].

The differences in Bayesian network was focused on the way in which they search through the space of nodes. In the process of searching, there are two steps: model evaluating and model optimization. There are many model evaluating methods such as Akaike Information Criterion (AIC), Minimum Description Length (MDL), and Cross-Validation Likelihood (CVL). In this paper, we adopted a simple estimator [42], as a fellow formula:

(4) $$P\left(x_{i}=k\;|\;pa\left(x_{i}\right)=j\right)=\frac{N_{ijk}+N_{ijk}^{\prime }}{N_{ij}+N_{ij}^{\prime }},$$

where *N*_*ijk*_ is 0.5 by default and sets the other.

For model optimization, we adopted K2 that one simple and very fast learning algorithm starts a given ordering of the features. Then it processes each node in turn and greedily considers adding edges from previously processed nodes to the current one. In each step it adds the edge that maximizes the network's score. When there is no further improvement, attention turns to the next node [41]. K2 uses the posteriori probability for estimating the structure of network:

(5) $$P\left(D\;|\;B_{s}\right)=\overset{n}{\underset{i=1}{\prod }}P\left(x_{i},pa\left(x_{i}\right)\right).$$

## 3. Results and Discussion

### 3.1. Symptoms Selection Based on Mingling Syndromes

Symptoms are selected to reduce the dimension of symptoms in predicting syndromes of CAD and to find the most related symptom subsets to improve the precision of syndrome prediction. In this experiment, datasets were grouped into three subsets: the TCM subset, the western subset, and the comprehensive subset. Every case was labeled with asthenia, sthenia or mingling syndrome. We collected 78 TCM symptoms in the TCM subset, 35 lab-measured indexes in the western medicine subset, and 113 mingling symptoms in the comprehensive subset. We quantitatively assessed the relatedness of each feature for syndrome prediction by SVM feature selection on the basis of tenfold cross-validation tests. By means of SVM feature selection, symptom ranking results of three subset sare shown in Table 2.

The performance of symptom selection was estimated by the classifier. In this experiment, we adopted seven classifiers: Naïve Bayes, Bayesian network, C4.5, Logistic, RBF Network, SMOSVM, and KNN. These seven classifiers are implemented in Weka [43, 44]. And parameters of classifiers are important in the processing of data mining. In our work, default parameters of software Weka are used. In general, the accuracy of the classifier is used to assess effectiveness of classification. However, in our dataset, the distribution of the three classes was not uniform. Consequently we adopted an integrative index to estimate the selected symptom subset. An ROC index was used for our experiment because it is insensitive to changes in class distribution and the ROC curves will not change if the proportion of positive to negative instances changes in the dataset [45–47]. The ROC curve is two two-dimensional graphs in which the true positives rate is plotted on the *y*-axis and the false positives rate is plotted on the *x*-axis. An ROC graph depicts relative tradeoffs between benefits and costs [45]. To compare classifiers, we may want to reduce ROC performance to a single scalar value representing expected performance. A common method is to calculate the area under the ROC curve (AUC) [45]. Multiclass problems are estimated by measuring AUC of every class, then summing the weighted AUC [45]:

(6) $${\text{AUC}}_{c}=\underset{c_{i}\in C}{\sum }\text{AUC}\left(c_{i}\right)\times p\left(c_{i}\right),$$

where AUC(*c*_*i*_) is the AUC of class *c*_*i*_, *p*(*c*_*i*_) is the distribution of class *c*_*i*_. 

**Figure 2:**
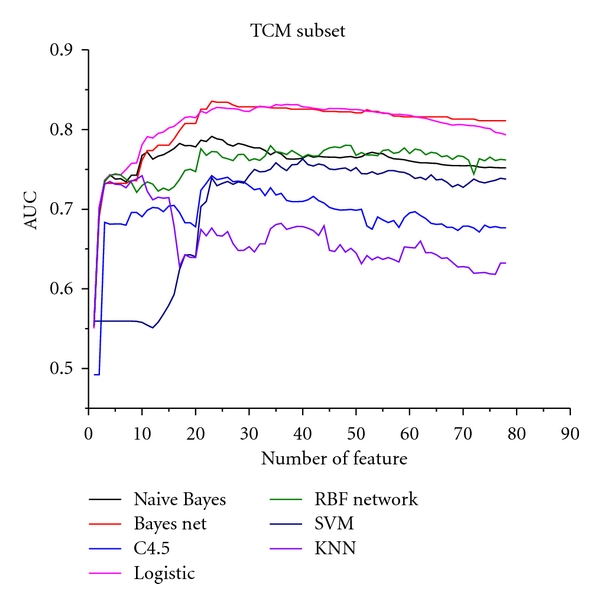
Relationship between AUC and symptom number in the TCM subset.

**Figure 3:**
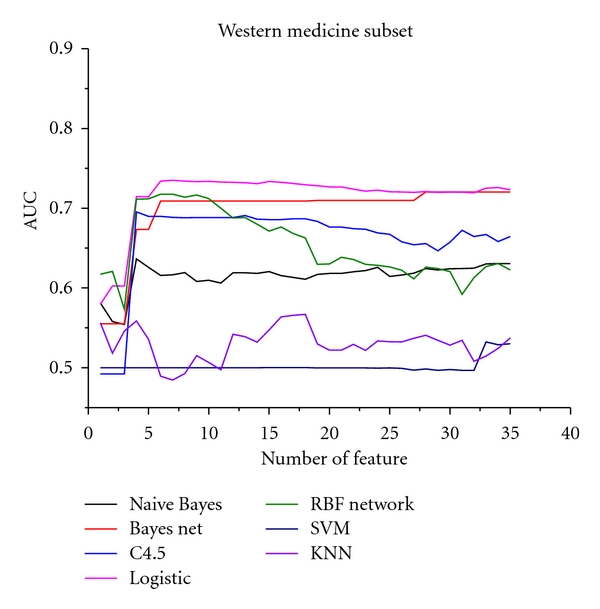
Relationship between AUC and symptom number in the western medicine subset.

**Figure 4:**
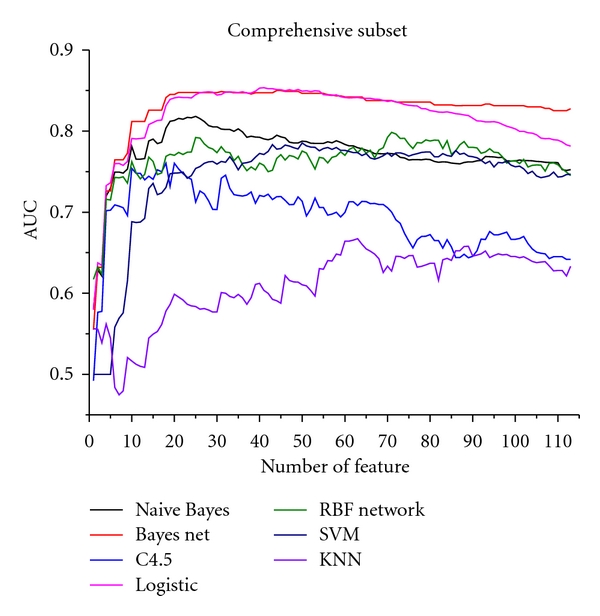
Relationship between AUC and symptom number in the comprehensive subset.

 The relationships between the AUC and symptom number in TCM subset are shown in Figure 2; Figure 3 is the western medicine subset; Figure 4 in the comprehensive subset. The horizontal coordinate is the weighted AUC with 1 as the highest value; the vertical coordinate represents the number of the feature.

**Figure 5:**
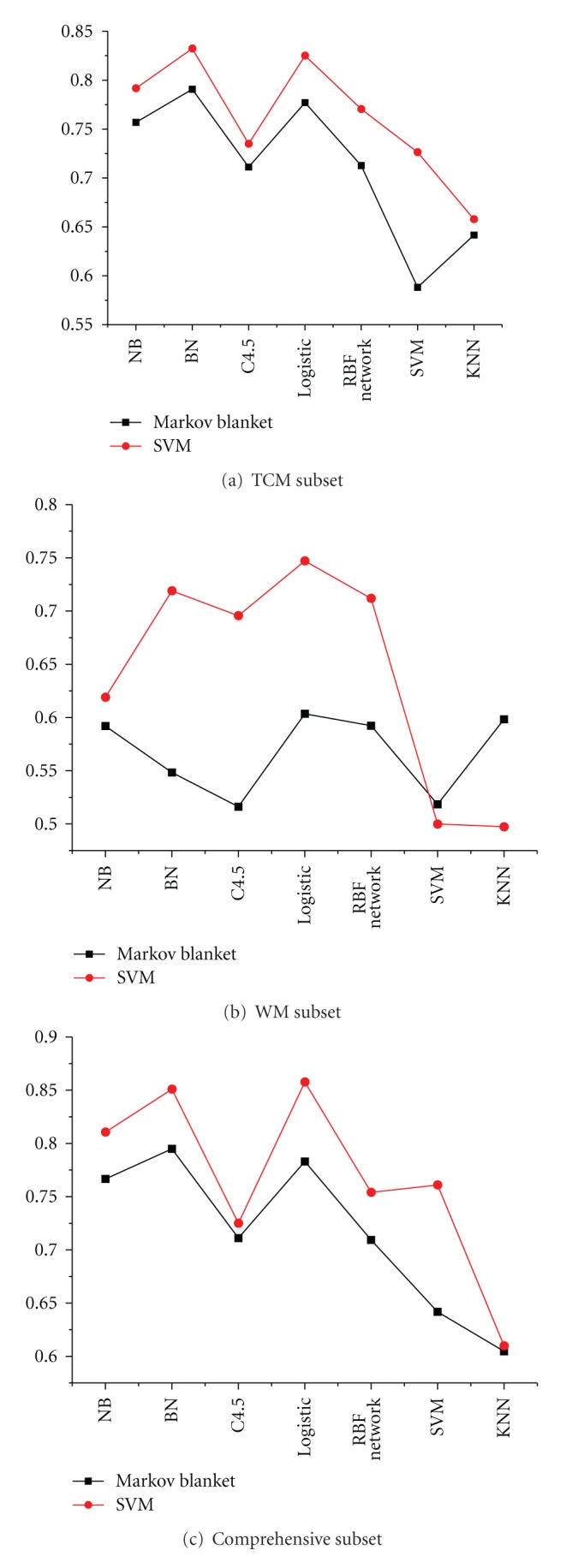
Comparative results of weighted AUC by using SVM and Markov blanket methods.

Compared with SVM feature selection, we also constructed the Markov blanket method, which considered the performance in the field of feature selection. After Markov blanket feature selection, we observed 28 symptoms in the TCM subset, 10 in the western medicine subset, and 35 in the comprehensive subset. We selected the top 25, 10, and 35 symptoms from the ranked list of three subsets. These results are shown in Figure 5. Results show that SVM feature selection has better performance than the Markov blanket feature selection from Figure 5.

**Figure 6:**
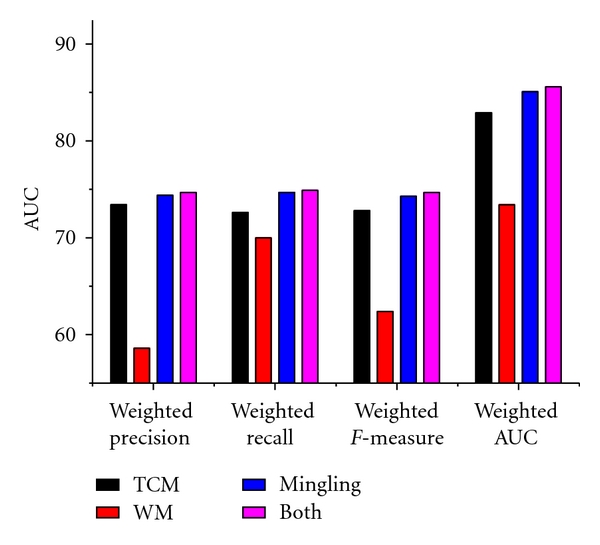
Comparative results of syndrome prediction using the Bayesian network classifier.

In all results, the optimum feature subset is essential to predict syndromes of CAD. From Figures 2, 3, and 4, the classification performance is optimum when 25 symptoms are selected from the TCM subset, 10 symptoms from the western subset, and 35 symptoms from the comprehensive subset. In the comprehensive subset, some critical symptoms (both TCM and western medicine) were filter. Therefore, we constructed a new subset selected from the optimum TCM and the western medicine subsets. We built four syndrome prediction models by using the Bayesian network classifier for the above four subsets-based on tenfold cross-validation test. Results are shown in Figure 6, which shows that the new constructing symptom subset performed better than the others. Lastly, we adopted the new constructing symptom subset as the featured set for predicting syndromes of CAD.

### 3.2. Results of Predicting Syndromes

All 35 symptoms above were collected for predicting syndromes of CAD. According with the foundational theory of TCM, sthenia can be divided into Qi stagnation, blood stasis, cold coagulation, phlegm turbidity, heat accumulation, water retention, and dampness pathogen, while asthenia can be divided into Qi deficiency, blood deficiency, Yin deficiency, Yang deficiency, heart deficiency, liver deficiency, spleen deficiency, kidney deficiency, and lung deficiency. In this paper, we constructed syndrome prediction models of Qi stagnation, blood stasis, cold coagulation, phlegm turbidity, heat accumulation, water retention, and dampness pathogen. On the dataset with the optimum symptoms, a prediction model of the Bayesian network was built as described in Section 2. Results are shown in Table 3, where the weighted precision is ∑_*c*_*i*_∈*C*_precision(*c*_*i*_) × *p*(*c*_*i*_), the weighted recall is ∑_*c*_*i*_∈*C*_recall(*c*_*i*_) × *p*(*c*_*i*_), the weighted *F*-Measure is ∑_*c*_*i*_∈*C*_ *f*measure(*c*_*i*_) × *p*(*c*_*i*_), and the weighted AUC is ∑_*c*_*i*_∈*C*_AUC(*c*_*i*_) × *p*(*c*_*i*_).

**Figure 7:**
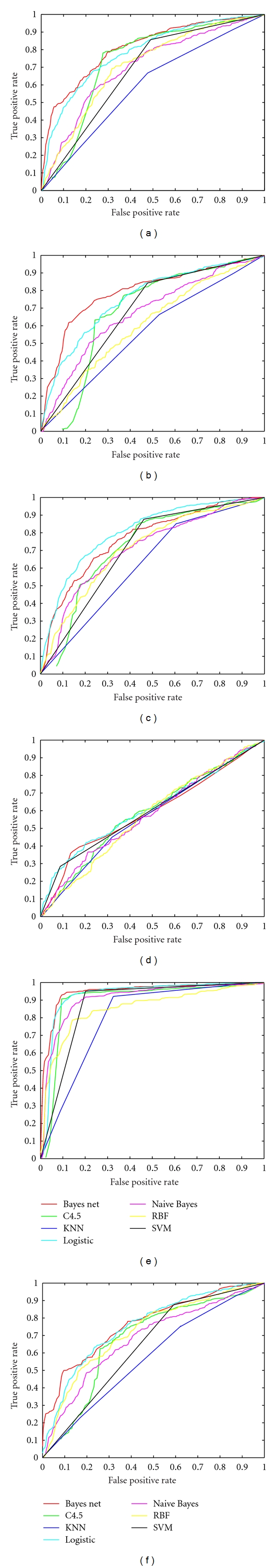
Comparative results of syndrome prediction with five classifiers.

We extensively compare the Bayesian network predictor with the following four methods: C4.5, Logistic, Naïve Bayes, and RBF network. And these five methods are implemented by Weka. Default parameters are exploited to predict syndromes. ROC curve analyses were used for estimating the performance of five classifiers. Comparative results are shown in Figure 7.

Figure 7 shows that the Bayesian network predictor achieved better performance than the others. Overall, these comparisons further demonstrate the feasibility and effectiveness of the Bayesian network classification approach for predicting syndromes of CAD.

## 4. Conclusion

In this paper, we attempted to predict patient syndromes according to our constructed predicting model based on the related symptoms separately in TCM and western medicine. Instead of using all of the symptoms in diagnosis, SVM feature selection can be used to select 35 of the 113 symptoms by assessing the predictive power of syndrome prediction. The prediction process implemented by feature selection techniques achieved more successful forecasting performance. In addition, they reduced the dimensions of the dataset so that the complexity of the syndrome predictor was decreased. The 35 symptoms subset was significant to diagnosis in clinical practice. Syndrome prediction processes of CAD based on the Bayesian network wasemployed to construct the prediction models of six syndromes for CAD in TCM. It resulted in better performance than four classifiers by means of ROC curve analyses without affecting the distribution of classes. We can conclude that our methods may be used for predicting the syndromes of CAD. Further research is under way addressing doctors' experience and knowledge related to constructing a Bayesian network structure.

## Figures and Tables

**Table 1:** Symptom list.

Symptoms of comprehensive subset					
Symptoms of TCM subset				Symptoms of western medicine	
(1) Chest pain	(21) Sighing	(41) Frothy sputum	(61) Red eye	(79) ST normal	(97) Ef
(2) Oppression in chest	(22) Depression	(42) Pharyngeal foreign body	(62) Deep-colored eye weeks	(80) ST lower than 0.1	(98) A/e
(3) Shortness of breath	(23) Inappetence	(43) Thirst without large fluid intake	(63) Eyelids swelling	(81) ST greater than 0.1	(99) Wall motion
(4) Palpitation	(24) Abdominal distension	(44) Tastelessness	(64) Dark red lip and gingivitis	(82) ST limb breast high	(100) Valve regurgitation
(5) Cough	(25) Ruffian of epigastrium	(45) Bitter taste in mouth	(65) Light-colored lip and methyl	(83) ECG	(101) Regurgitant degree
(6) Chilly sensation and the cold limbs	(26) Belching	(46) Sweet taste in mouth	(66) Deep-colored palate mucosa	(84) Q wave	(102) Leukocyte
(7) Tiredness and fatigue	(27) Nausea and vomiting	(47) Salty taste in mouth	(67) Less abdominal pressure	(85) Frequent extrasystole	(103) Neutral %
(8) Spontaneous sweating	(28) Loose stool	(48) Sticky and greasy sensation in mouth	(68) Lower extremity edema	(86) High left ventricular voltage	(104) Lymph %
(9) Night sweating	(29) Constipation	(49) Morning diarrhea	(69) Faint low voice	(87) T wave	(105) Erythrocyte
(10) Dysphoria with feverish sensation in chest, palms, and soles	(30) Soreness and weakness of waist and knees	(50) Powerless in defecation	(70) Atrophy	(88) Diameter of main root	(106) Hemoglobin
(11) Dry eyes	(31) Frequent urination at night	(51) Deep-colored urine	(71) Tongue quality	(89) Main pulmonary	(107) Platelet
(12) Dry mouth	(32) Limb numbness	(52) Clear urine in large amounts	(72) Patchy petechia and ecchymosis	(90) Left atrial dimension	(108) Fasting plasma glucose
(13) Dizziness	(33) Heel pain	(53) Residual urine	(73) Tongue body	(91) Interventricular septum thickness	(109) TG
(14) Amnesia	(34) Hemiplegic limbs	(54) Coldness in abdomen and waist	(74) Quality of tongue coating	(92) Pulsatile range	(110) TG
(15) Vertigo	(35) Subcutaneous ecchymosis	(55) Heavy limbs	(75) Color of tongue coating	(93) End-diastolic diameter	(111) HDL
(16) Tinnitus	(36) Rough skin	(56) Pale complexion	(76) Body fluid on tongue coating	(94) Systolic diameter	(112) LDL
(17) Facial flush	(37) Obesity	(57) Suddenly white complexion	(77) Vein color	(95) Right ventricular diameter	(113) Fibrinogen
(18) Insomnia	(38) White phlegm	(58) Darkish complexion	(78) Vein type	(96) Outflow tract	
(19) Fussy temper and irascibility	(39) Yellow phlegm	(59) Sallow complexion			
(20) Distending pain in the hypochondria	(40) Blood in the sputum	(60) Flushing			

**Table 2:** Ranked symptoms by means of SVM feature selection.

Dataset	Rank list of NO. symptom
TCM	75, 8, 73, 52, 36, 50, 22, 54, 40, 31, 13, 26, 30, 42, 23, 74, 71, 6, 49, 27, 7, 25, 78, 11, 20, 35, 4, 60, 34, 65, 10, 72, 33, 32, 59, 63, 9, 3, 67, 61, 57, 17, 18, 66, 64, 43, 5, 45, 76, 19, 38, 77, 16, 24, 2,28, 14, 44, 62, 56, 70, 55, 1, 68, 53, 29, 21, 12, 37, 47, 39, 58, 15, 69, 48, 46, 51, 41

WM	17, 27, 26, 30, 13, 20, 18, 15, 11, 29, 16, 14, 12, 10, 7, 35, 33, 24, 22, 31, 5, 28, 34, 25, 19, 23, 4, 9, 32, 8, 3, 6, 1, 2, 21

Comprehensive	95, 71, 102, 108, 92, 78, 107, 101, 73, 7, 97, 40, 27, 8, 82, 22, 85, 75, 31, 23, 74, 109, 103, 42, 30, 5, 10, 35, 106, 50, 6, 52, 65, 11, 57, 20, 89, 18, 13, 81, 113, 111, 79, 77, 36, 54, 9, 104, 67, 60, 44, 25, 72, 64, 83, 16, 3, 59, 24, 32, 21, 49, 26, 55, 4, 63, 33, 43, 88, 99, 84, 66, 28, 68, 17, 45, 80, 34, 38, 70, 14, 94, 76, 37, 51, 62, 110, 100, 86, 112, 61, 48, 87, 1, 2, 90, 39, 91, 53, 41, 96, 56, 19, 47, 69, 46, 15, 58, 12, 93, 105, 29, 98

**Table 3:** Results of syndrome prediction based on Bayesian network.

	Index			
Syndrome	Weighted precision	Weighted recall	Weighted *F*-Measure	Weighted AUC
Blood stasis	0.763	0.761	0.762	0.811
Phlegm turbidity	0.740	0.746	0.742	0.791
Qi deficiency	0.750	0.747	0.748	0.766
Yin deficiency	0.656	0.663	0.640	0.589
Yang deficiency	0.926	0.926	0.926	0.946
Kidney deficiency	0.735	0.728	0.731	0.766
